# A comparison between protein profiles of B cell subpopulations and mantle cell lymphoma cells

**DOI:** 10.1186/1477-5956-7-43

**Published:** 2009-11-23

**Authors:** Henrik Stranneheim, Lukas M Orre, Janne Lehtiö, Jenny Flygare

**Affiliations:** 1 Department of Gene Technology, AlbaNova University Center, Royal Institute of Technology, Stockholm, Sweden; 2 Karolinska Biomics Center, Karolinska University Hospital and Department of Oncology and Pathology, Karolinska Institutet, Stockholm, Sweden; 3 Department of Laboratory Medicine, Division of Pathology, Karolinska University Hospital Huddinge, F-46, SE-14186 Stockholm, Sweden

## Abstract

**Background:**

B-cell lymphomas are thought to reflect different stages of B-cell maturation. Based on cytogenetics and molecular markers, mantle cell lymphoma (MCL) is presumed to derive predominantly from naïve, pre-germinal centre (pre-GC) B lymphocytes. The aim of this study was to develop a method to investigate the similarity between MCL cells and different B-cell compartments on a protein expression level.

**Methods:**

Subpopulations of B cells representing the germinal centre (GC), the pre-GC mantle zone and the post-GC marginal zone were isolated from tonsils using automated magnetic cell sorting (AutoMACS) of cells based on their expression of CD27 and IgD. Protein profiling of the B cell subsets, of cell lines representing different lymphomas and of primary MCL samples was performed using top-down proteomics profiling by surface-enhanced laser detection/ionization time-of-flight mass spectrometry (SELDI-TOF-MS).

**Results:**

Quantitative MS data of significant protein peaks (p-value < 0.05) separating the three B-cell subpopulations were generated. Together, hierarchical clustering and principal component analysis (PCA) showed that the primary MCL samples clustered together with the pre- and post-GC subpopulations. Both primary MCL cells and MCL cell lines were clearly separated from the B cells representing the GC compartment.

**Conclusion:**

AutoMACS sorting generates sufficient purity to enable a comparison between protein profiles of B cell subpopulations and malignant B lymphocytes applying SELDI-TOF-MS. Further validation with an increased number of patient samples and identification of differentially expressed proteins would enable a search for possible treatment targets that are expressed during the early development of MCL.

## Introduction

In the last decade, several studies of lymphomas have been performed using microarray analysis of global gene expression [1]. Genomic profiling has proven to be of both diagnostic and prognostic value. However, the correlation between mRNA and protein expression is generally poor, and regulation of many physiological processes is post-transcriptional. Protein analysis is therefore essential for the elucidation of biological mechanisms behind e.g. lymphoma formation. Proteomic profiling using liquid chromatography-MS/MS has been applied to a limited number of lymphomas of different origin [2]. The high throughput top-down method of SELDI-TOF-MS, which is based on chip-binding of intact proteins and endogenous peptides, has been used to differentiate follicular lymphoma from Burkitt's lymphoma [3] and to distinguish between diffuse large B-cell lymphoma, follicular lymphoma and mantle cell lymphoma (MCL) [4]. This method is optimal for the detection of peptides or small proteins in the mass range of 1000-25000 Daltons [5]. Many proteins and peptides that have regulatory roles in proliferation, differentiation, apoptosis and cell signaling are found within this range.

**Figure 1:**
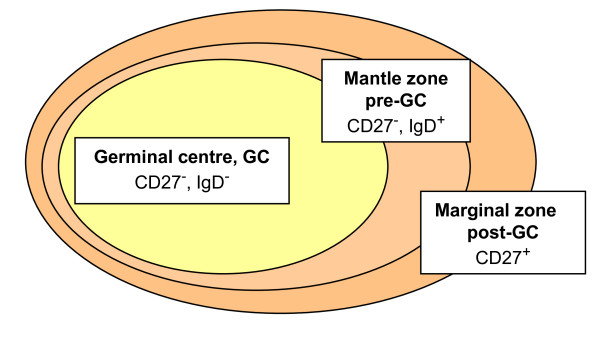
**The three major B cell compartments in peripheral lymphoid organs**. Presence of the differentiation markers CD27 and IgD in each zone of a B cell follicle is indicated.

Each stage in the B cell maturation process is characterized by a particular structure of the B cell receptor and expression patterns of differentiation markers, e.g. the pre-germinal centre (GC) marker IgD or the post-GC marker CD27 (Fig. 1). During the process, which involves double-stranded DNA breaks and rearrangements, there is a potential for malignant transformation. The differentiation markers can therefore be used to characterize different human B cell lymphomas, that appear to be "frozen" at the different development stages [6].

We have applied this approach in the comparison of MCL cells with subpopulations of normal B lymphocytes. MCL is a relatively rare malignancy that represents 5-10% of all non-Hodgkins lymphomas [7]. Although several genetic and molecular mechanisms involved in the pathogenesis of MCL have been defined, there is currently no effective standard treatment for the disease, and the clinical outcome is poor [7,8]. While most human B-cell lymphomas are derived from cells at the GC or post-GC stage of development [9], MCL has been thought to originate from naïve, pre-GC B cells [10]. However, approximately 20% of the MCL cells, which are abundant in the mantle zone of lymphoid follicles [11], have undergone somatic hypermutations in the immunoglobulin variable heavy-chain genes, suggesting a heterogeneous origin [10].

In the present study, we used SELDI-TOF-MS to generate expression profiles of intact proteins and peptides. MCL cells and cell lines representing other B cell malignancies were compared with subpopulations of B-cells representing the three major compartments of peripheral lymphoid organs; the GC, the pre-GC mantle zone and the post-GC marginal zone.

## Materials and methods

### Ethical permission

This study was approved by the Ethics committee at Karolinska Institutet, Etikkommitté syd, #689/03.

### Cell lines and primary MCL

The MCL cell lines Jvm-2 [12,13], Rec-1 [14,15], Granta 519 [16] and JeKo-1 [17], the precursor leukemia cell line Nalm-6 [18] representing the pre-GC stage, the Burkitt's lymphoma cell lines Radji [19] and Namalwa [20] representing GC and the plasma cell line SKMM-2 [21,22] representing post-GC were used in the study. All cell lines were tested for mycoplasma and were found to be free from infection. The tests were performed with MycoAlert Mycoplasma Detection Kit (LT07-218, Cambrex, USA) on a Thermo Luminoskan Ascent (Thermo Fischer Scientific, Inc, USA). The cell lines were cultured in RPMI 1640 GlutaMax^tm^-1 (72400-021, Invitrogen, USA), supplemented with 10% FBS (16140-071, Invitrogen, USA) and 50 μg/mL gentamicin (15750-045, Invitrogen, USA) at 37°C using a 5% CO_2 _atmosphere, except JeKo-1, which was cultured using a 20% FBS supplement. The cells were harvested in the exponential phase, snap frozen in liquid nitrogen and stored as pellets at -70°C. In addition, two primary MCL tumors, with confirmed t(11;14)(q13;q32) cyclin D1 translocation and immunophenotype of CD5^+^/CD23^-^, indicative of MCL [8], were included in the study. The primary MCL tumours contained >80% tumor cells, as verified using the CD5 and CD23 markers in a FACScan (BD, USA) (not shown). Biological triplicates were used for the primary cells except MCL tumours, that were analyzed in duplicate due to lack of availability of samples.

### Isolation of B cell populations from healthy individuals

The normal B cell populations were extracted from fresh tonsils obtained from patients undergoing tonsillectomies at the surgical department, Karolinska University Hospital, Huddinge. The tonsils were dissected and then homogenized using a Medimachine (Dako Denmark A/S, Glostrup, Denmark). The resulting cell suspension was purified from erythrocytes by treatment with a lysis buffer containing ammonium chloride for 8 minutes and subsequent filtration to remove cellular debris. The B cell population was separated from T cells by negative selection. The pan T cell CD3^+ ^surface receptor was conjugated with magnetic anti-CD3^+ ^microbeads (130-050-101, Miltenyi Biotec, Germany), which were sorted by an AutoMACS sorter (Miltenyi Biotec, Germany) using column 130-021-101 (Miltenyi Biotec, Germany). The purity of the T and B cell fractions was validated by using the pan B cell surface receptor CD19^+ ^[6] and the T cell CD3^+ ^receptor. The B cell fractions were >90% CD19^+ ^as determined by incubating with fluorescent CD3^+^/FITC + CD19^+^/RPE antibodies (FR866, DAKO, Denmark) and analyzing with a FACScan (BD, USA) (not shown). The total B cell extracts were snap frozen in liquid nitrogen and stored as pellets at -70°C.

### Isolation of B cell subpopulations representing pre-GC, GC and post-GC

In order to separate pre-GC, GC and post-GC populations from the tonsils, the total B cell populations from the AutoMACS separation were incubated with magnetic CD27^+ ^microbeads (130-051-601, Miltenyi Biotec, Germany), generating a post-GC CD27^+ ^population and a CD27^- ^population consisting of both pre-GC and GC B cells. The CD27^- ^population was then further sorted using an anti-IgD^+^-FITC antibody and Anti-FITC microbeads (130-048-701, Miltenyi Biotec, Germany), to isolate the pre-GC and GC fractions.

### Protein preparation and concentration determination

Cell pellets from cell lines and isolated primary B cell populations were subjected to a lysis buffer (150 mM NaCl, 50 mM Tris-HCL pH 7,5, 0,1% Triton X-100, 1% CHAPS) and repeatedly immersed in liquid nitrogen to lyse the cells and release the proteins. Protein concentrations were determined using a BCA protein assay kit (23225, PIERCE, USA) according to the manufacturer's instructions.

### Analysis of proteins using SELDI-TOF

The samples were dissolved to a final concentration of 0.15 ug/ul in 50 mM ammonium acetate pH 4.5 and 0.1% Triton X-100. 100 μl of the prepared protein solutions were analyzed using a cation exchange (CM 10, pH 4,5) protein-chip (Biorad Inc., USA) [23]. Standard protocols recommended by the manufacturer were used in the experiments. Mass spectra were collected using SELDI-TOF Protein Biology System IIC using three different laser intensity and time lag focusing settings creating three data sets; 3-6 kDa, 6-10 kDa and 10-40 kDa (optimization of M/Z regions and of time lag focusing). The design of the experimental protocol was based on pilot experiments, to optimize peak number and minimize detector saturation from intense peaks at 5 kDa and 9 kDa. The data analysis was performed using CE software package Ciphergen Biosystems, CA, USA. All spectra were baseline corrected and peak detection was performed using signal/noise value of 3 and a valley depth of value of 2 (first pass).

### Statistical analysis

The significant differentially expressed protein peaks were identified using nonparametric t-test, using in pair-wise comparison Mann-Whitney and in group-wise comparison Kruskal Wallis test. The peaks with p-value < 0,05 were considered as significant differentially expressed proteins peaks.

### Clustering of protein profiles

Unsupervised hierarchical clustering of the primary populations and cell lines was performed using all peaks detected within each mass range. To distinguish between pre-GC, GC and post-GC populations, all peaks detected within the three populations which had p-value < 0,05 were considered significant and were used to cluster these primary populations within each data set. The corresponding peaks in the primary MCL samples and MCL cell lines were then included to generate a hierarchical clustering of MCL samples within the pre-GC, GC and post-GC populations. The peaks were analyzed by heat map and principal component analysis (PCA). Ciphergen Express software, Ciphergen, CA, USA, was used for both hierarchical clustering and PCA.

## Results

### Isolation of B cell subpopulations representing pre-GC, GC and post-GC

**Figure 2:**
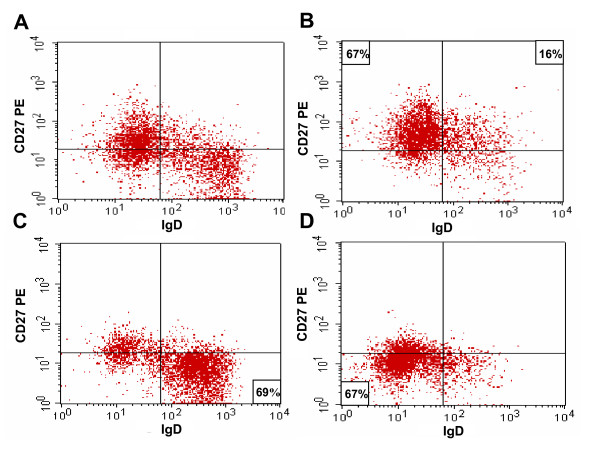
**FACScan images showing the different sub-populations of B cells before and after AutoMACS sorting**. (A) The total B cell population before CD27 separation. (B) The CD27^+ ^population representing post-GC. (C) TheCD27^-^/IgD^+ ^population representing pre-GC. (D) The CD27^-^/IgD^- ^population representing GC.

B lymphocytes were isolated from tonsils by negative AutoMACS selection and elimination of T cells expressing the CD3^+ ^surface receptor. The purity of the B cell fraction was >90% as determined using the CD19^+ ^B cell marker (data not shown). In order to isolate viable subpopulations of B lymphocytes representing different stages of the maturation process, the total B cell population was incubated with magnetic CD27^+ ^microbeads. Histograms of the cells before (Fig. 2A) and post-sorting (Fig. 2B-D) are shown. The AutoMACS separation resulted in a CD27^+ ^population representing post-GC cells (Fig. 2B), and a CD27^- ^population consisting of both pre-GC and GC B cells. Using the anti-IgD^+^-FITC antibody and targeting anti-FITC microbeads to the fluorophore of the primary antibody, the IgD^+ ^pre-GC (Fig. 2C) and IgD^- ^GC (Fig. 2D) populations could be separated. This procedure generated viable cells with a purity of 67% in the CD27^- ^IgD^- ^population, 69% in the CD27^- ^IgD^+ ^population and 83% (67+16%) in the CD27^+ ^population (Fig. 2).

### Hierarchical clustering and principal component analysis of primary cells and cell lines

**Figure 3:**
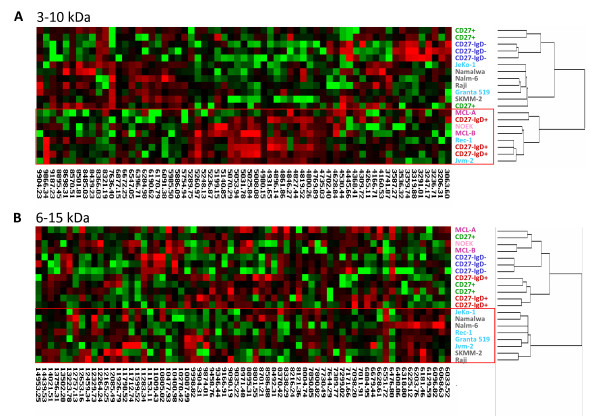
**Hierarchical clustering of B cell sub-populations, cell lines and MCL patient samples**. The three subpopulations are labeled as follows: CD 27^+ ^(green, post-GC), CD 27^-^/IgD^+ ^(red, pre-GC) and CD 27^-^/IgD^- ^(blue, GC). The two primary MCL samples are depicted in dark pink, and the cultured primary MCL cells NOEK in light pink. The MCL cell lines are depicted in turquoise and the remaining cell lines in grey. Clustering within the (A) 3-10 kDa and (B) 6-15 kDa ranges.

In order to clarify the possible similarity between MCL cells and the purified subpopulations of B cells representing pre-GC, GC and post-GC cells, protein expression profiling was performed. Hierarchical clustering is an unsupervised method that allows data to self-organize according to similarity between samples. In this study the method was applied with respect to protein expression pattern. The different stages of B cell maturation were represented by the AutoMACS-sorted B-cell subpopulations and by the precursor leukemia cell line Nalm-6 (pre-GC), the Burkitt's lymphoma cell lines Raji and Namalwa (GC) and the plasma cell line SKMM-2 (post-GC). MCL was represented by two primary MCL samples, MCA A and MCL B, and the MCL cell lines Jvm-2, Rec-1, Granta 519 and JeKo-1. In addition, a sample denoted NOEK was analyzed. This consisted of cells derived from MCL B that had been kept in culture under the same conditions as the other cell lines. Within the 3-10 kDa range, the samples clustered into two main groups (Fig 3A, upper panel). One of the groups contained the primary MCL samples, NOEK and the MCL cell lines Rec-1 and Jvm-2 together with the B cell subpopulations representing pre-GC. The remaining cell lines and B cell subpopulations clustered together in the other group. Except for Rec-1 and Jvm-2, the cell lines did not cluster according to their presumed origin. This phenomenon was even more pronounced in the 6-15 kDa range (Fig. 3B, lower panel), where the cell lines and the primary B lymphocytes formed separate clusters.

**Figure 4:**
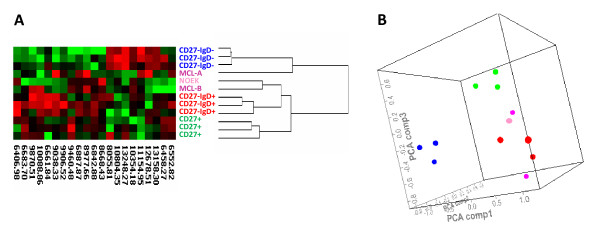
**Hierarchical clustering and principal component analysis within the 6-15 kDa area of peaks that significantly (p-value < 0.05) separated the B cell sub-populations**. (A) Cluster analysis of the three subpopulations CD 27^+ ^(green, post-GC), CD 27^-^/IgD^+ ^(red, pre-GC) and CD 27^-^/IgD^- ^(blue, GC). The two primary MCL samples (dark pink) and the cultured primary MCL cells NOEK (light pink) were included in the analysis. (B) PCA plot representing the same samples and peaks as in (A).

**Figure 5:**
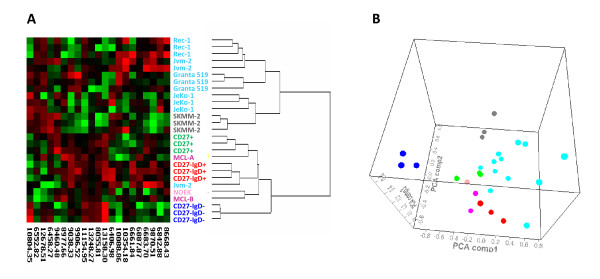
**Hierarchical clustering and principal component analysis within the 6-15 kDa area of peaks that significantly (p-value < 0.05) separated the B cell sub-populations**. (A) Cluster analysis of the three subpopulations CD 27^+ ^(green, post-GC), CD 27^-^/IgD^+ ^(red, pre-GC) and CD 27^-^/IgD^- ^(blue, GC). In addition to the two primary MCL samples (dark pink) and the cultured primary MCL cells NOEK (light pink), MCL cell lines (turquoise) and a plasma cell line (grey) were included in the analysis. (B) PCA plot representing the same samples and peaks as in (A).

In order to increase the sensitivity and specificity of the analysis, peaks that significantly (p-value < 0.05) separated the primary pre-GC, GC and post-GC populations were selected. The analysis was performed in the 6-15 kDa area, since the number of peaks in the 3-10 kDa area was too low using this approach. In the subsequent analysis, the subpopulations representing pre- and post-GC clustered more closely to each other than to the GC subpopulations (Fig. 4A). The primary MCL sample MCL-B and the cultured primary MCL cells NOEK clustered together with the pre-GC subpopulations, while MCL-A clustered together with the GC subpopulation (Fig. 4A). However, in PCA, a cluster comprising the pre-GC subsets and all MCL samples was observed (Fig. 4B). When cell lines were included in the hierarchical clustering, all MCL cell lines except one triplicate of Jvm-2 clustered together with the plasma cell line SKMM-2 (Fig. 5a). Similar to the results in Fig. 3b, the cell lines used in this analysis formed a cluster that was separate from the cluster comprising the primary cells. MCL-B, NOEK and one of the Jvm-2 triplicates clustered together with the pre-GC subsets, while MCL-A clustered together with the post-GC subpopulations (Fig. 5a). The primary MCL clustered together with the pre- and post-GC subsets in PCA, while both MCL cell lines and primary MCL were clearly separated from the GC subpopulation (Fig. 5b).

## Discussion

In the present study, we used AutoMACS sorting in order to isolate subpopulations of B cells reflecting the pre-GC, GC and post-GC stage. In the subsequent analysis of protein expression, the cation exchange protein-chip array CM 10 was used at pH 4,5. A previous study including MCL samples has shown that the same chip surface resulted in detection of the highest number of peaks [4]. In the initial analysis, the primary MCL samples A and B clustered together with the pre-GC subpopulations in the 3-10 kDa range. Two of the MCL cell lines, Rec-1 and Jvm-2, clustered together with the primary MCL, while Granta 519 and JeKo did not. Using global gene expression analysis, the MCL cell lines Granta 519, SP53 and NCEB1 have earlier been shown to cluster closely to primary MCL samples [24]. Overall, the other cell lines did not cluster together with the B cell subpopulations they were supposed to represent, based on their origin. In the 6-15 kDa range, all of the cell lines clustered together, indicating the presence of proteins that show similar expression levels exclusively in the cell lines, despite their different origin. One factor influencing the expression pattern could be cell culture conditions. However, the NOEK population of primary MCL cells that had been cultured under identical conditions to the MCL cell lines for one week did not cluster together with them. Neither is EBV-transformation likely to account for the similarity of the cell lines in the 6-15 kDa range, as both the JeKo-1 and Rec-1 cell lines, which clustered together with the other cell lines, are EBV-negative. This observation indicates that studies based on cell lines must be interpreted with caution and emphasize the need to analyze patient samples in order to understand the biology behind diseases. In an extensive gene expression study using 60 cell lines of different origin, it has earlier been shown that the majority of the cell lines were poor representatives of their cellular origin [25].

The subpopulations of the biological triplicates from tonsil that were labeled with the same set of antibodies clustered together when peaks that significantly distinguished the subpopulations (p-value < 0,05) were analyzed following SELDI-TOF-MS. Thus, the purity of the subpopulations following AutoMACS sorting was sufficient to generate reproducible protein profiles from each stage. The GC-population clustered separately from the pre-GC and post-GC populations. This could reflect up-regulation of the expression of specific proteins required for GC maturation and down-regulating of others. When separating the post-GC CD27^+ ^population using CD27^+ ^microbeads, the post-GC fraction contained mostly CD27^+^/IgD^-^, but also 10-25% CD27^+^/IgD^+ ^B cells representing early memory cells. IgD is a pre-GC marker, and the presence of IgD^+ ^early memory cells might also influence the clustering together of the pre-GC and post-GC populations.

In the hierarchical clustering and PCA of peaks that significantly (p < 0.05) distinguished the B cell subsets, the primary MCL samples and the cultured primary MCL cells NOEK clustered together with the pre- and post-GC subpopulations. The similarity between MCL cells and pre-GC cells is in accordance with the prevailing assumptions about the pathology of MCL [10]. However, the present study needs to be expanded in order to determine if proteome profiling using SELDI-TOF-MS supports a pre-GC or heterogeneous origin of MCL cells. Interestingly, the GC subpopulations were clearly separated from both the primary MCL samples and the MCL cell lines in PCA. Thus, it appears as if the biology of MCL cells differs from that of B cells residing in the GC compartment.

In conclusion, this pilot study has shown that purifying B lymphocytes using AutoMACS-sorting combined with profiling of intact proteins using SELDI-TOF-MS allows a comparison to be made between the protein expression of MCL cells and subpopulations of B-cells representing the three major compartments of peripheral lymphoid organs. Further studies based on a larger patient cohort and followed by MS-based identification of proteins may contribute to the understanding of the molecular pathogenesis of MCL and other B cell malignancies, and conceivably suggest new potential treatment targets.

## Competing interests

The authors declare that they have no competing interests.

## Authors' contributions

HS carried out the preparation of primary cells, the cell culturing, cell sorting and preparation of proteins. HS and LMO carried out the SELDI-TOF analysis, hierarchical clustering and PCA. JL and JF participated in the design and supervision of the study. JL performed the statistical analysis. JF drafted the manuscript. All authors read and approved the final manuscript.
